# Vaginal Mesh Revision Surgery: A Focus on Functional Outcomes

**DOI:** 10.3390/jcm15186982

**Published:** 2026-09-09

**Authors:** Rui Pedrosa, Carlos Marquez, Manuel Saavedra Centeno, Clara Velasco Balanza, Lira Pelari, Javier Casado, Luis San-José, Luis López-Fando

**Affiliations:** 1 Department of Urology and Renal Transplantation, Unidade Local de Saúde Coimbra, 3004-561 Coimbra, Portugal; 2 Department of Urology, Hospital Universitario de la Princesa, 28006 Madrid, Spain; carlosmarquezguemez@gmail.com (C.M.); msaavedrac97@gmail.com (M.S.C.); clara.velasco@salud.madrid.org (C.V.B.); lira.pelari@salud.madrid.org (L.P.); javi.casado.varela@gmail.com (J.C.); lsanjoseman@gmail.com (L.S.-J.); llfando@gmail.com (L.L.-F.); 3 Clinica UROLF, C. de Joaquín Costa, 28, Chamartín, 28002 Madrid, Spain; 4 Catedra de Uroginecología, Universidad Alfonso X, 28691 Madrid, Spain

**Keywords:** urology, complications, mesh, surgery, functional outcomes

## Abstract

**Background/Objectives**: Mesh-related complications after surgery for stress urinary incontinence (SUI) and pelvic organ prolapse (POP) remain a clinical challenge, and surgical revision is often required. However, its impact on functional outcomes and continence status is incompletely defined. This study aimed to evaluate voiding and continence outcomes after surgical revision of vaginal synthetic mesh. **Methods**: This retrospective multicenter study included 55 consecutive women undergoing surgical revision of synthetic mesh for symptomatic complications after SUI or POP procedures between March 2021 and April 2025. Preoperative evaluation included clinical assessment, uroflowmetry, and selective imaging or urodynamic studies. Surgical management included mesh section, partial removal, or total removal via vaginal, abdominal, or combined approaches. The primary outcome was change in Qmax and post-void residual (PVR). Secondary outcomes included symptom resolution, changes in urgency urinary incontinence (UUI) and SUI, and postoperative complications. **Results**: Obstructive voiding dysfunction was the leading indication for revision (47.3%). Median Qmax increased from 13.0 to 18.0 mL/s (*p* = 0.0013), and median PVR decreased from 55.0 to 5.0 mL (*p* < 0.001). UUI improved from 56.4% to 34.5% (*p* = 0.017, McNemar’s exact test). Conversely, SUI increased from 20.0% to 56.4% (*p* < 0.001), although only 16 patients (29.1%) had clinically significant postoperative SUI, of whom 7 underwent a secondary anti-incontinence procedure. Intraoperative complications were infrequent (5.5%), while postoperative urinary tract infections occurred in 16.4%. **Conclusions**: Mesh revision surgery improves voiding function and reduces storage symptoms in selected patients but carries a substantial risk of recurrent or de novo SUI, underscoring the need for careful patient selection and counseling, with an acceptable safety profile in experienced centers.

## 1. Introduction

The surgical management of female stress urinary incontinence (SUI) and pelvic organ prolapse (POP) has evolved significantly over recent decades, with the widespread adoption of synthetic mesh-based techniques. Mid-urethral slings (MUS) have become the gold standard for SUI due to their high efficacy and minimally invasive nature [1,2], while transvaginal mesh (TVM) was historically used for POP repair [3] before its use became increasingly restricted.

Despite their initial success, the growing use of synthetic mesh has been accompanied by an increasing recognition of mesh-related complications. These include vaginal exposure, extrusion, erosion into adjacent organs such as the bladder or urethra, chronic pelvic pain, dyspareunia, and voiding dysfunction, which may significantly impair quality of life [4,5]. Regulatory actions, including those from the U.S. Food and Drug Administration, have further highlighted safety concerns and led to the withdrawal of several transvaginal mesh products from the market [6].

The management of mesh-related complications remains complex and challenging. While conservative approaches may be appropriate for selected cases, many patients require surgical revision, ranging from mesh incision to partial or complete removal [5]. These procedures are technically demanding due to the fibrotic reaction and altered anatomical planes induced by synthetic materials, increasing the risk of intraoperative complications.

A key clinical dilemma in this setting is the balance between symptom resolution and preservation of functional outcomes. While mesh removal may relieve obstruction, pain, or erosion-related symptoms, it may also compromise urethral support, leading to recurrence or de novo stress urinary incontinence [7,8,9]. Despite the large number of mesh procedures performed worldwide, high-quality data on functional outcomes following revision surgery—particularly in real-world settings—remain limited.

The aim of this study was to evaluate the clinical presentation, surgical management, and functional outcomes of women undergoing mesh revision surgery for complications related to SUI and POP repairs.

## 2. Materials and Methods

This retrospective multicenter study included 55 consecutive women, identified from the surgical databases of two specialized pelvic floor referral centers, who underwent surgical revision of a mid-urethral sling (MUS), transvaginal mesh (TVM), or combined implant for symptomatic mesh-related complications between March 2021 and April 2025. The study is reported in accordance with the STROBE statement for observational studies.

Symptomatic complications were defined as bothersome voiding symptoms, recurrent urinary tract infections attributed to mesh-related obstruction, vaginal mesh exposure or extrusion, pelvic pain, or dyspareunia considered clinically related to the mesh. Patients managed conservatively, with incomplete data, or with follow-up shorter than 12 months were excluded.

Preoperative evaluation included clinical history, pelvic examination, and functional assessment. Pelvic examination incorporated POP-Q staging when applicable, cough stress test for objective SUI, and assessment of urethral mobility by Q-tip test. A fixed urethra was defined as urethral axis rotation < 30°. Uroflowmetry (Qmax, voided volume, and post-void residual [PVR]) was performed in all patients. Additional investigations, including translabial ultrasound, cystoscopy, and invasive urodynamics, were performed as clinically indicated. Urodynamic studies followed International Continence Society standards. Bladder outlet obstruction was defined by the female bladder outlet obstruction index (BOOI = PdetQmax − 2.2 × Qmax) > 5 [10]; detrusor overactivity as any involuntary detrusor contraction during filling; and detrusor underactivity as PdetQmax < 20 cm H_2_O with Qmax < 15 mL/s and bladder voiding efficiency < 90%. Surgical approach was individualized according to the type and location of the mesh. A vaginal approach was preferred for suburethral or vaginally accessible mesh, while laparoscopic or robotic-assisted approaches were reserved for retropubic, intravesical, or complex cases. A combined approach was used when necessary. No standardized institutional algorithm governed the extent of revision, which was individualized according to the presenting complication, the anatomical location and integrity of the implant, the degree of tissue integration, and the anticipated risk of injury to the urethra, bladder or adjacent organs. Surgical revision was classified as mesh section (division without removal), performed for isolated obstructive dysfunction related to a suburethral sling; partial removal (removal of the affected segment), principally for the suburethral portion of the implant; or total removal (removal of the entire mesh), for extensive exposure, intraluminal migration, or involvement of a larger prosthetic implant.

All patients had a minimum follow-up of 12 months, with evaluations at 3, 6, and 12 months; the 3- and 6-month visits served for clinical monitoring and detection of early complications, while the 12-month visit was the pre-specified endpoint for all functional outcomes reported. The primary outcome was change in objective voiding parameters (Qmax and PVR). Secondary outcomes included resolution of the presenting complication, change in urgency urinary incontinence (UUI), recurrence or de novo SUI, need for further treatment, and postoperative complications, classified according to the Clavien–Dindo system. Continuous variables were assessed for normality (Shapiro–Wilk) and are presented as mean ± SD or median (IQR), as appropriate, while categorical variables are expressed as frequencies and percentages. Paired comparisons were performed using Wilcoxon signed-rank tests for continuous variables and McNemar’s exact test for paired binary outcomes. All tests were two-sided, with *p* < 0.05 considered statistically significant.

## 3. Results

### 3.1. Baseline Characteristics

A total of 55 women were included in the analysis. Median age was 65 years (IQR 57–72) and median body mass index (BMI) was 26.0 kg/m^2^ (IQR 24.0–27.9). Most patients were postmenopausal (87.3%, *n* = 48). The most common comorbidities were arterial hypertension (40.0%, *n* = 22) and dyslipidemia (34.5%, *n* = 19). On physical examination, relevant findings included objective stress urinary incontinence (20.0%), fixed urethra (54.5%), and vaginal mesh extrusion (25.5%). Detailed findings, including POP-Q staging, are summarized in Table 1.

### 3.2. Preoperative Evaluation

Preoperative assessment revealed a predominance of obstructive functional patterns. Median Qmax was 13 mL/s (IQR 10.0–17.5) and median post-void residual (PVR) was 55 mL (IQR 4.0–101.0), with 27.3% of patients presenting PVR > 100 mL. Invasive urodynamic studies were performed in 46 patients (83.6%) and demonstrated bladder outlet obstruction in 34.8%, detrusor overactivity in 45.7%, and detrusor underactivity in 26.1%. Translabial ultrasound, performed in 31 patients (56.4%), revealed a malpositioned or folded mesh in 83.9%. Cystoscopic evaluation (*n* = 55) was unremarkable in most patients (81.8%), with relevant findings including urethral obstruction (10.9%) and mesh erosion or extrusion into the urinary tract (7.3%). These findings are detailed in Table 2.

The median time from primary surgery to mesh revision was 87 months (IQR 46–168). Mid-urethral slings accounted for most implants (72.8%), predominantly transobturator tapes (TOT), followed by retropubic tapes (TVT) and mini-slings. Transvaginal mesh for POP was identified in 14.5% of cases, while 12.7% had combined prosthetic configurations (mid-urethral slings plus mesh for POP).

The most frequent indication for revision was obstructive voiding dysfunction (47.3%), followed by vaginal mesh extrusion (25.4%), malpositioned mesh (14.5%), intraluminal migration involving either the bladder or the urethra (7.3%), and chronic pelvic pain (5.5%) (Table 3).

### 3.3. Surgical Outcomes and Intraoperative Findings

The surgical approach was determined by the anatomical location of the mesh and the nature of the complication. A vaginal approach was utilized in 83.6% of the cases. For more complex scenarios, including high retropubic erosions or extensive pelvic mesh, laparoscopic or robotic-assisted surgery was performed (10.9%), while 5.5% required a combined abdominovaginal approach.

Regarding the extent of surgical intervention, mesh section was performed in 15 cases (27.3%), partial removal in 30 cases (54.5%), and complete removal in 10 cases (18.2%).

Intraoperative complications were observed in three patients (5.5%). These included one vaginal opening, one intraperitoneal bladder perforation, and one case requiring bilateral JJ stent placement. All events were managed intraoperatively without long-term sequelae.

In terms of surgical setting, 27.3% of procedures were performed on an outpatient basis, while 72.7% required inpatient admission. Among hospitalized patients, most (87.5%) were discharged on postoperative day 1, with prolonged stays up to day 4 observed in five cases. The median duration of indwelling catheterization was one day, although 16.4% of patients required catheterization for more than 2 days. Postoperative morbidity included urinary tract infections in 16.4% of patients (*n* = 9), all managed with culture-directed antibiotics. No cases of urinary fistula were observed.

### 3.4. Postoperative Functional Outcomes

**Figure 1:**
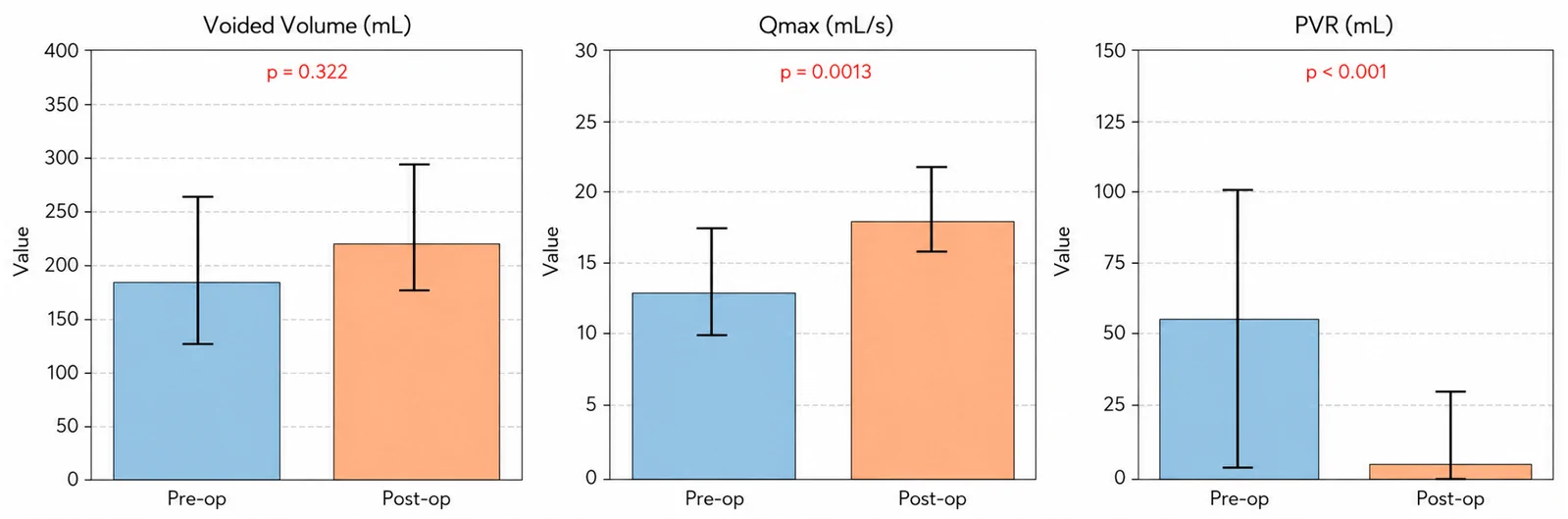
Changes in uroflowmetry parameters before and 12 months after mesh revision surgery.

Objective functional parameters improved significantly following surgical revision (Table 4 and Figure 1). Median maximum flow rate (Qmax) increased from 13.0 mL/s preoperatively to 18.0 mL/s at follow-up (Wilcoxon test, *p* = 0.0013), while median post-void residual (PVR) decreased from 55 mL to 5 mL (Wilcoxon test, *p* < 0.001). No significant change was observed in median voided volume (*p* = 0.322). These findings reflect a significant resolution of obstructive voiding dysfunction.

In parallel, storage symptoms improved, with urgency urinary incontinence (UUI) decreasing from 56.4% (31/55) preoperatively to 34.5% (19/55) at 12 months (McNemar’s exact test, *p* = 0.017), reflecting resolution in 17 of 31 affected women and de novo UUI in 5 of 24. Conversely, removal of suburethral support was associated with recurrence or de novo onset of stress urinary incontinence (SUI), with prevalence rising from 20.0% (11/55) to 56.4% (31/55) (*p* < 0.001). However, only 16 of these 31 patients met criteria for clinically significant SUI, defined as patient-reported bothersome incontinence confirmed by a positive cough stress test at follow-up evaluation.

Among the 16 patients with clinically significant postoperative SUI, 4 underwent implantation of a female artificial urinary sphincter (AUS), and 3 were treated with periurethral bulking agents. Four patients were considered candidates for AUS placement but had not yet undergone surgery at the time of manuscript preparation. The remaining 5 patients were lost to follow-up after the 12-month assessment, before any treatment for their SUI was initiated.

## 4. Discussion

The present study provides a real-world evaluation of women undergoing surgical revision for mesh-related complications after procedures for stress urinary incontinence (SUI) and pelvic organ prolapse (POP). Three findings stand out: first, obstructive voiding dysfunction was the leading indication for revision; second, mesh revision was associated with improvement in objective voiding parameters; and third, there was a substantial risk of recurrent or de novo SUI after removal of suburethral support.

The preoperative findings in our series highlight the heterogeneity of mesh-related morbidity. Patients frequently showed low flow, elevated post-void residual, abnormal urodynamic patterns, and imaging or endoscopic abnormalities, rather than a single uniform presentation. This is in line with current guideline recommendations, which emphasize structured evaluation with pelvic examination, selective cystoscopy, imaging, and urodynamics according to symptoms and suspected anatomical involvement [2].

The main strength of the present study lies in the functional postoperative data. After revision surgery, Qmax improved significantly and post-void residual decreased markedly, while voided volume remained stable, indicating that the principal effect of surgery was restoration of voiding efficiency rather than bladder capacity change. These findings are concordant with the recent study by Costa et al., who reported significant postoperative improvement in voiding parameters after delayed sling incision or removal for chronic voiding dysfunction [8], and with earlier revision series demonstrating high rates of resolution of obstructive symptoms after sling incision, with cure of voiding dysfunction reported in up to 97% of cases [11]. Similarly, the systematic review by Carter et al. concluded that surgical management of mesh complications is generally effective for alleviating urinary dysfunction, although outcome reporting across studies remains markedly heterogeneous, limiting direct comparison between series [7]. In parallel with the objective improvement in emptying, urgency urinary incontinence decreased in our cohort, consistent with the recognized clinical association between outlet obstruction and storage symptoms in women with mesh-related voiding dysfunction.

The counterbalance to this functional gain is the postoperative continence outcome. In our study, SUI prevalence rose from 20.0% preoperatively to 56.4% postoperatively, and 16 patients developed clinically significant postoperative SUI. This is one of the most important findings of the study and is highly consistent with the available literature. Six et al. reported SUI recurrence in 46.4% of women within 3 months of mid-urethral sling revision, with recurrence rates ranging from 7.7% after simple sling section to 66.7% after complete excision—a gradient directly related to the extent of tissue removed [12]. A large recent registry analysis similarly confirmed that the extent of mesh removal is one of the strongest predictors of continence failure after revision surgery, reinforcing the need for individualized, stepwise surgical decision-making rather than routine complete excision [13]. At the same time, not every woman with postoperative leakage required immediate or definitive reintervention. Of the 16 women with clinically significant SUI, 4 underwent artificial urinary sphincter implantation and 3 were treated with bulking agents, while 4 were considered candidates for sphincter placement but had not yet been operated. This distinction between any postoperative SUI and clinically significant SUI is relevant for counseling and for interpretation of revision outcomes.

The need for secondary anti-incontinence procedures after revision has also been described in the literature, with Six et al. reporting subsequent surgery in 21.8% of patients [12]. In our series, 12.7% of the cohort underwent a secondary procedure, rising to 20.0% if the four women awaiting sphincter implantation are included.

Regarding surgical strategy, most revisions in our cohort were performed vaginally, with minimally invasive abdominal approaches reserved for more complex retropubic, intravesical, or extensive pelvic cases. This distribution mirrors an emerging trend in the literature, where robotic-assisted approaches are increasingly favored for retropubic or intravesical mesh, offering improved visualization and more complete excision compared with vaginal techniques, particularly for eroded or migrated mesh [14]. This is consistent with current guideline-based practice, in which the route and extent of excision are guided by the type of complication and the anatomical location of the implanted material [2].

The overall complication rate in our cohort was low. By comparison, Rac et al. reported that 47.3% of patients experienced at least one complication following mesh excision, although the majority were minor (Clavien–Dindo grade I–II) and resolved without long-term sequelae [15]. In our series, complications were similarly infrequent and without long-term sequelae, with postoperative morbidity mainly related to urinary tract infections. Although UTI is not consistently reported as a separate outcome across mesh revision studies, it remains a recognized and clinically relevant postoperative complication that merits standardized reporting in future series.

This study has some limitations. The main one is the absence of validated patient-reported outcome measures: mesh-related complications carry a subjective burden that objective parameters cannot capture, and our results should therefore be read as evidence that revision restores voiding efficiency, not that it restores quality of life. This contrasts with series using instruments such as the UDI-6 [16], which have shown that patient-perceived success after mesh removal does not always align with objective parameters, with a substantial proportion of women still reporting recurrent SUI or urgency despite anatomically successful excision. Incorporating such measures in future prospective studies would allow for a more complete assessment of the true impact of revision surgery on quality of life. The retrospective design may also introduce selection bias, and cystoscopy and urodynamic studies were performed selectively according to clinical indication, which may have introduced ascertainment bias. The extent of excision was determined by surgeon judgment rather than standardized criteria and was driven by the indication, so this study was not designed to compare outcomes between surgical techniques. Most primary implants had been placed at external institutions and the original operative reports were largely unavailable, so the device model, technical details of implantation, and surgeon-related variables could not be retrieved.

Taken together, these findings suggest that the anatomical and functional heterogeneity of mesh-related complications warrants an individualized surgical strategy, tailored to the extent and location of the implant rather than a uniform approach to excision. Future prospective studies incorporating validated patient-reported outcome measures and longer follow-up will be needed to better define the trade-off between symptom relief and continence preservation, and to identify predictors that could guide the extent of mesh removal on a patient-by-patient basis.

## 5. Conclusions

Mesh revision surgery is an effective treatment for selected patients with symptomatic complications following SUI and POP procedures, with significant improvement in objective voiding parameters and reduction in storage symptoms. However, this benefit came at the cost of stress urinary incontinence in 56.4% of women; incontinence was clinically significant in 29.1% and led to a secondary anti-incontinence procedure in 12.7%. These procedures should therefore be regarded not as simple removal operations, but as reconstructive decision-making that weighs symptom relief against a risk of continence impairment that aggregate SUI rates tend to overstate. Careful patient selection and thorough preoperative counseling remain essential, and, when performed in experienced centers, mesh revision demonstrated an acceptable overall safety profile.

## Figures and Tables

**Table 1:** Preoperative physical examination findings. The table summarizes the anatomical findings recorded during the physical assessment prior to mesh revision surgery.

Findings		Frequency—*n* (%)
Pelvic Organ Prolapse (POP) (POP-Q Staging)	No POP	33 (60.0%)
	Grade I	12 (21.8%)
	Grade II	7 (12.7%)
	Grade III/IV	3 (5.5%)
Stress Urinary Incontinence	Objective SUI (Cough Test)	11 (20.0%)
	Negative Cough Test	44 (80.0%)
	Fixed Urethra	30 (54.5%)
Mesh Complications	Vaginal Extrusion	14 (25.5%)

**Table 2:** Summary of preoperative diagnostic workup findings.

	Finding	Result
Uroflowmetry (*n* = 55)	Median Qmax (mL/s)	13.0 (IQR 10.0–17.5)
	Median Post-void Residual (PVR, mL)	55.0 (IQR 4.0–101.0)
Cystoscopy (*n* = 55)	No abnormal findings	45 (81.8%)
	Urethral obstruction	6 (10.9%)
	Bladder erosion/Urethral extrusion	3 (5.5%)/1 (1.8%)
Translabial Ultrasound (*n* = 31)	Malpositioned or Folded Mesh	26 (83.9%)
Invasive Urodynamic Study (*n* = 46)	Bladder Outlet Obstruction (BOO)	16 (34.8%)
	Detrusor Overactivity (DO)	21 (45.7%)
	Detrusor Underactivity (DU)	12 (26.1%)

**Table 3:** Indications for mesh revision in the study population.

Indication for Revision	Frequency—*n* (%)
Obstructive Voiding (and Complications)	26 (47.3%)
Vaginal Mesh Extrusion	14 (25.5%)
Malpositioned Mesh	8 (14.5%)
Intraluminal Migration (Vesical/Urethral)	4 (7.3%)
Chronic Pelvic/Vaginal Pain	3 (5.5%)

**Table 4:** Comparison of preoperative and 12-month postoperative uroflowmetry parameters (*n* = 55; paired studies available for all patients).

Parameter	Preoperative Median (IQR)	Postoperative Median (IQR)	*p*-Value (Wilcoxon Test)
Voided Volume (mL)	184.0 (130.0–264.5)	222.0 (180.0–294.5)	0.322
Maximum Flow Rate (Qmax) (mL/s)	13.0 (10.0–17.5)	18.0 (16.0–22.0)	0.0013
Post-Void Residual (PVR) (mL)	55.0 (4.0–101.0)	5.0 (0.0–28.0)	<0.001

## Data Availability

The data presented in this study are available on request from the corresponding author due to privacy and ethical restrictions related to patient confidentiality.

## Abbreviations

The following abbreviations are used in this manuscript:

SUI	Stress urinary incontinence
POP	Pelvic organ prolapse
UUI	Urgency urinary incontinence
PVR	Post-void residual
MUS	Mid-urethral sling
TVM	Transvaginal mesh
